# Supplementary material for: Loss of synaptopodin impairs mGluR5 and protein synthesis–dependent mGluR-LTD at CA3-CA1 synapses
Source: PNAS Nexus. 2024 Feb 8;3(2):pgae062. doi: 10.1093/pnasnexus/pgae062 (PMC10879843; doi:10.1093/pnasnexus/pgae062)
Supplement: pgae062_Supplementary_Data [file pgae062_supplementary_data.pdf]

Figure S1

A

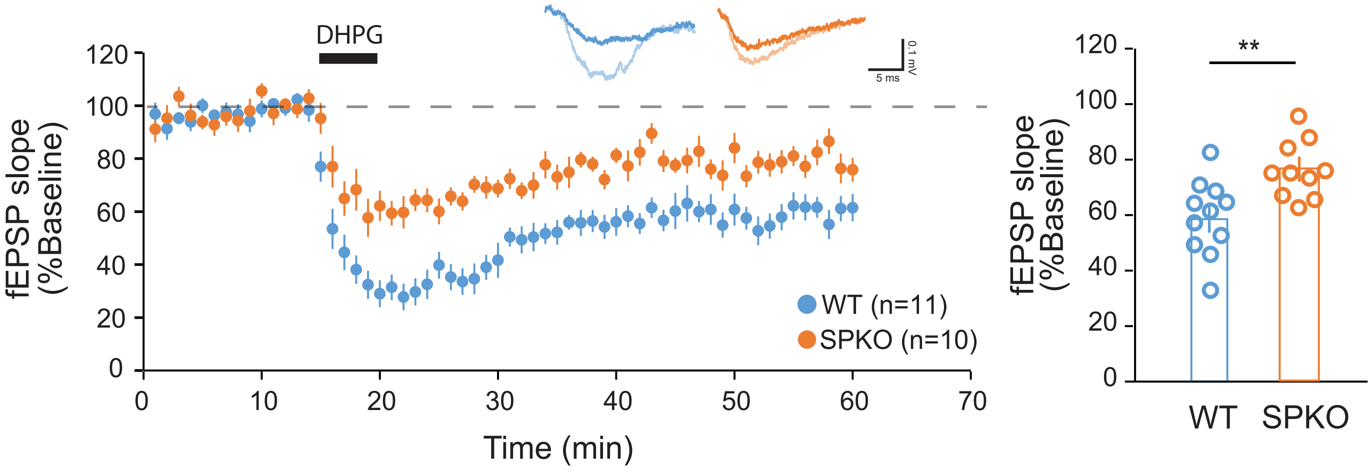
DHPG
0.1 mV
5 ms
fEPSP slope (%Baseline)
120
100
80
60
40
20
0
0
10
20
30
40
50
60
70
Time (min)
WT (n=11)
SPKO (n=10)
**
WT
SPKO

Supplementary Figure legends

Figure S1: mGluR-LTD is significantly impaired in SPKO mice compared to WT. (*Left)* Time course of normalized fEPSP in WT (n=11 slices, N=9 mice) and SPKO (n=10 slices, N=7 mice). (*Right)* Quantification of average mGluR-LTD in the last 10 min of the recording. Data are mean ± SEM. WT=59.1%±4.8, SPKO=76.5%±4.4. **p<0.01 (Mann-Whitney test).
